# Phenotypic novelty in experimental hybrids is predicted by the genetic distance between species of cichlid fish

**DOI:** 10.1186/1471-2148-9-283

**Published:** 2009-12-04

**Authors:** Rike B Stelkens, Corinne Schmid, Oliver Selz, Ole Seehausen

**Affiliations:** 1 Division of Aquatic Ecology & Macroevolution, Institute of Ecology and Evolution, University of Bern, Baltzerstr. 6, CH-3012 Bern, Switzerland; 2 Department of Fish Ecology and Evolution, Centre for Ecology, Evolution and Biogeochemistry (CEEB), Eawag Swiss Federal Institute of Aquatic Science and Technology, Seestrasse 79, CH-6047 Kastanienbaum, Switzerland; 3 Department of Ecology and Evolution, University of Lausanne, Biophore, CH-1015 Lausanne, Switzerland

## Abstract

**Background:**

Transgressive segregation describes the occurrence of novel phenotypes in hybrids with extreme trait values not observed in either parental species. A previously experimentally untested prediction is that the amount of transgression increases with the genetic distance between hybridizing species. This follows from QTL studies suggesting that transgression is most commonly due to complementary gene action or epistasis, which become more frequent at larger genetic distances. This is because the number of QTLs fixed for alleles with opposing signs in different species should increase with time since speciation provided that speciation is not driven by disruptive selection. We measured the amount of transgression occurring in hybrids of cichlid fish bred from species pairs with gradually increasing genetic distances and varying phenotypic similarity. Transgression in multi-trait shape phenotypes was quantified using landmark-based geometric morphometric methods.

**Results:**

We found that genetic distance explained 52% and 78% of the variation in transgression frequency in F1 and F2 hybrids, respectively. Confirming theoretical predictions, transgression when measured in F2 hybrids, increased linearly with genetic distance between hybridizing species. Phenotypic similarity of species on the other hand was not related to the amount of transgression.

**Conclusion:**

The commonness and ease with which novel phenotypes are produced in cichlid hybrids between unrelated species has important implications for the interaction of hybridization with adaptation and speciation. Hybridization may generate new genotypes with adaptive potential that did not reside as standing genetic variation in either parental population, potentially enhancing a population's responsiveness to selection. Our results make it conceivable that hybridization contributed to the rapid rates of phenotypic evolution in the large and rapid adaptive radiations of haplochromine cichlids.

## Background

Interspecific hybridization as an evolutionary force has a mixed chronicle in the literature. Despite important early work [1-6], the image of hybridization in evolutionary literature has only recently changed from that of a predominantly destructive force to a more balanced view, giving due credit to hybridization as a potential catalyst of phenotypic evolution and indeed diversification. Not only have cases of hybrid speciation been demonstrated conclusively both in plants and animals [7-11], but hybridization is now implicated in the generation of whole adaptive radiations in plants [12,13], animals [14-16] and prokaryotes [17] (reviewed in [18,19]). Besides the general surge of genetic variation ensuing from the admixture of divergent genomes [20], and the acquisition of specific adaptive traits through lateral gene transfer [17,21] and introgression [22-24], another potential outcome of hybridization that may facilitate adaptive diversification into new directions is the occurrence of qualitatively or quantitatively novel phenotypes referred to as transgressive segregation. Transgression describes the phenomenon that segregation variance in hybrid offspring can result in phenotypes with extreme trait values exceeding the range of parental trait values in either the positive or negative direction [25,26]. Agricultural breeding programs have long benefited from transgressive phenotypes as a means to improve cultivars but studying the adaptive potential of transgression in evolutionary research is only a recent development. Transgression can in principle affect any quantitative trait and has been demonstrated for morphological traits (skull morphology of cichlid fish: [27]), physiological traits (salt tolerance in *Helianthus *sunflowers: [28]), life history traits (flowering time in *Arabidopsis*: [29]), and behavioural traits (mating behaviour of *Drosophila*: [30]). For instance, Parnell et al. [31] recently described a mechanism whereby hybridizing cichlid species with different morphologies but similar functions are likely to produce functionally transgressive progeny.

Previous work on the genetic basis of transgression indicates that it is most often caused by the action of complementary genes between QTL loci that carry alleles of opposite signs in the parents but sum up to larger or smaller trait values compared to the parents when combined in a hybrid genome [22,29,32-39]. One interesting prediction emerging from this, especially put forward by Rieseberg et al. [25], is that the amount of transgression should increase as a function of the genetic distance between the parental lines. This is because the number of loci for which the parents have fixed alleles with opposite effects should increase with time since isolation during the divergence of species, which would thus result more frequently in complementary gene action.

Besides genetic distance, transgression is predicted to also be affected by the phenotypic similarity of the parents [25]. Transgression and phenotypic differentiation have been suggested to be inversely correlated such that phenotypically similar species produce more transgressive hybrid offspring than phenotypically dissimilar parents [22,33,36,40]. This is because large phenotypic differences between two species may often result from divergent directional selection, a process expected to eventually lead to the fixation of alleles with the same sign across all QTL within a species, and mostly opposite signs between the species. This would produce F1 offspring heterozygous at most of these loci. Although some F2 progeny may then have QTL combinations that could exhibit complementary gene action, this will unlikely produce transgressive trait values. In other words, during evolution under divergent selection, opportunity for transgression decreases due to a loss of the required kind of genetic variation. Conversely, if the parents show rather similar phenotypes, despite considerable genetic distance, this indicates the action of stabilizing selection. The genetic basis for transgression is then more likely given because stabilizing selection leads to alternating fixation of alleles with negative and positive trait values, and the sequence of fixation of alleles with either sign at different QTLs will by chance be different between isolated populations. In agreement with this prediction, a study on transgression in hybrids between two cichlid fish species revealed novel phenotypes only in traits with a selection history other than consistent directional selection [27]. To the extent that phenotypic and genetic divergence between species are correlated, the effects of phenotypic differentiation can potentially confound or cancel out the predicted relationship between genetic distance and transgression [25,40].

Despite the knowledge of the genetic basis of transgression, tests on the effects of genetic and phenotypic distance on transgressive segregation remain inconclusive [41-43], mostly because the few existing studies covered only small or unknown ranges of genetic distance and were not designed to test the two predictions introduced above. Only recently, a comparative study [40] using data on plant and animal hybrids found evidence that distantly related species more often produce hybrids with extreme trait values than closely related species.

Here, we produced seven interspecific crosses using African haplochromine cichlid fish covering a wide range of pairwise genetic distances and phenotypic distances. We set out to test 1) if transgression occurred in F1 and F2 hybrids, 2) if the amount of transgression was predictable from genetic distance between the parental species, and 3) if transgression was predictable from the phenotypic differentiation between the parental species. We raised F1 hybrids, F2 hybrids, and the corresponding homospecific control crosses until sexual maturity under controlled laboratory conditions. The amount of transgressive segregation per cross type was quantified using landmark-based geometric morphometric methods and a thin-plate spline procedure. Genetic distances between parental species were estimated using mitochondrial D-loop sequences from GenBank and three different molecular clocks were applied to convert distances into absolute divergence time. Multi-trait phenotypic distances between the parental species were estimated using Mahalanobis distances calculated from geometric morphometric data.

## Results

### Transgressive segregation in hybrids

Thirty F1 hybrid families from seven different cross types and three families of each of the nine homospecific crosses were obtained (see Table 1 for number of families and number of individuals per cross type). Transgressive phenotypes were found in all hybrid cross types (Additional file 1) albeit not in every family (Table 2). On average, F1 hybrids exceeded the phenotypic range of the parental species by 14% ± 13% (± standard deviation). This average was calculated across all cross types and across all axes of shape variation, weighted by the percent variance each axis explained.

**Table 1:** The nine homospecific crosses and seven different interspecific hybrid crosses used to measure transgressive segregation with their geographical origin and the number of families per cross type.

cross type	homospecific crosses		n families (n individuals per family)	origin (lake/rivers)
1	*Pundamilia nyererei (P. ny)*		3 (33, 35, 8)	Victoria
2	*Pundamilia pundamilia (P. pun)*		3 (27, 15, 16)	Victoria
3	*Neochromis omnicaeruleus (N. omni)*		3 (7, 5, 30)	Victoria
4	*Paralobidochromis rockkribensis (P. rock)*		3 (18, 27, 18)	Victoria
5	*Paralobidochromis chilotes (P. chil)*		3 (17, 4, 16)	Victoria
6	*Metriaclima estherae (M. est)*		3 (29, 23, 5)	Malawi
7	*Astatotilapia burtoni (A. burt)*		3 (11, 16, 16)	Tanganyika and rivers
8	*Astatotilapia calliptera (A. call)*		3 (38, 48, 27)	Malawi and rivers
9	*Protomelas taeniolatus (P. taen)*		3 (9, 26, 22)	Malawi

**cross type**	**hybrid crosses**		**n families (n individuals per family)**	

	**male parent**	**female parent**	**F1 hybrids**	**F2 hybrids**
1	*Neochromis omnicaeruleus*	*Pundamilia pundamilia*	4 (21,29,45,33)	4 (5,10,19,9)
2	*Paralobidochromis chilotes*	*Pundamilia nyererei*	2 (24,19)	5 (19,21,2,3,8)
3	*Paralobidochromis rockkribensis*	*Pundamilia pundamilia*	3 (37,26,43)	-
4a	*Astatotilapia calliptera*	*Metriaclima estherae*	5 (3,11,11,9,6)	8 (2,22,16, 4, 6, 7,4,7)
4b	*Metriaclima estherae*	*Astatotilapia calliptera*	3 (2,21,16)	4 (4,12,11,10)
5	*Protomelas taeniolatus*	*Astatotilapia calliptera*	2 (21,43)	7 (12,12,13,4,14,5,12)
6	*Astatotilapia burtoni*	*Astatotilapia calliptera*	4 (6,2,19,19)	5 (9,10,15,17,16)
7	*Pundamilia nyererei*	*Astatotilapia. calliptera*	8 (15,20,5,15,20,30,22,28)	8 (4,18,4,1,9,4,6,17)

The number of photographed and measured individuals per family is shown in brackets. Sex-reversed crosses of the same cross type are indicated by 'a' and 'b'.

**Table 2:** All hybrid crosses with pairwise genetic distances (uncorrected p-distance calculated from mitochondrial D-loop sequences), divergence times (in millions of years based on two different relaxed molecular clocks and the internally calibrated clock) and phenotypic shape differentiation based on Mahalanobis distances.

Cross type	Species crossed	Genetic distance	Divergence time internal clock (lower-upper bound)	Divergence time fossil record	Divergence time Gondwana break-up	Phenotypic distance	*% T*_*total*_	**Transgressive families **(%)	*% T*_*total*_	**Transgressive families **(%)
							**F1 hybrids**		**F2 hybrids**	
								
1	*N. omni × P. pun*	0.007^1^	0.35-0.61	0.10	0.14	5.69	12.55	75	14.73	100
2	*P. chil × P. ny*	0.007^1^	0.35-0.61	0.10	0.14	13.23	30.76	100	6.42	80
3	*P. rock × P. pun*	0.007^1^	0.35-0.61	0.10	0.14	3.95	32.48	100	-	-
4	*M. est × A. call*	0.0188	0.93-1.64	0.58	0.92	16.19	0.14	37.5	18.20	71.4
5	*P. taen × A. call*	0.0241	1.19-2.1	0.89	1.49	19.29	3.71	100	14.40	100
6	*A. burt × A. call*	0.0408	2.02-3.56	2.23	4.12	22.12	5.58	50	39.07	100
7	*P. ny × A. call*	0.0553	2.74-4.82	3.78	7.43	7.09	13.9	62.5	32.46	75

The total amount of transgression (*T*_*total*_) occurring on the major axes of phenotypic shape variation is shown separately for F1 and F2 hybrids. Also reported is the proportion of transgressive families obtained per cross type (i.e. the number of families containing transgressive phenotypes divided by the total number of families of that cross type).

^1^Note that these distance estimates are likely overestimates of genetic distance between species. Distances are based on sequence differences between mitochondrial D-loop haplotypes, but these species have highly incomplete haplotype lineage sorting. Hence any distance obtained from a small sample of sequences is likely to overestimate species distance

**Figure 1:**
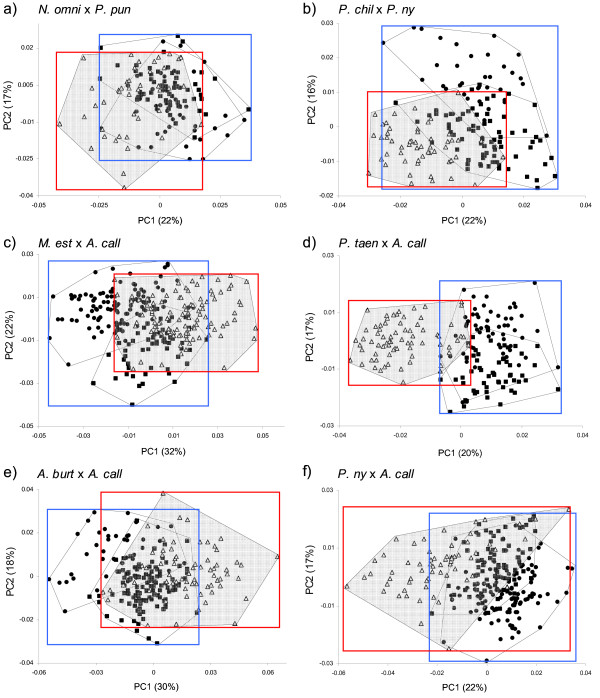
**Results of principal component analyses using geometric morphometrics data to quantify the amount of transgression in shape of interspecific hybrids of haplochromine cichlids**. Graphs show the distribution in morphospace of six different F2 hybrid crosses and the corresponding homospecific crosses of species pairs with increasing genetic distance from smallest (a, b) to largest distance (f). Abbreviations of species names correspond to Table 1. Every data point represents one individual. Filled symbols indicate parental species, triangles indicate F2 hybrids. Blue squares encompass the phenotype range of the combined parental species; red squares represent the phenotype range of F2 hybrids. The percentage of variance explained by principal component 1 and 2 are shown in brackets. Note that the visualization of transgression is restricted to the first two axes of shape variation here, which is not (or not entirely) representative of the total amount of transgression found per cross type.

Forty-one F2 hybrid families from six different cross types were obtained (Table 1). Transgression was observed in all cross types (Figure 1) albeit not in every family (Table 2). F2 hybrids exceeded the phenotypic range of the parental species on average by 21% ± 12%. The amount of transgression and variance explained by each PC axis for both F1 and F2 hybrids is shown in detail in Additional file 2.

In all F1 and F2 hybrid cross types, there were significant differences between families in the distribution of phenotypes in morphospace. MANOVAs with family as factor and all relevant PCs as response variables suggested that within each cross type, at least one hybrid family was significantly different from another family along at least one axis of shape variation (all test results including the number of PCs used per MANOVA are shown in Additional file 3). However, transgression analysis revealed that on average 75% of all F1 families (2-5 families per cross type) and 84% of all F2 families (3-7 families per cross type) contained transgressive phenotypes demonstrating that transgressive segregation was not caused by single-family effects (Table 2).

### Transgressive segregation as a function of genetic distance

Uncorrected pairwise p-distances between species pairs, calculated from D-loop sequences, ranged from 0.007 to 0.055. Depending on the molecular clock used, this translates into a range of absolute time since speciation from several thousand years to 2.7/3.8/7.4 million years (internal/fossil record/Gondwana fragmentation calibration; from here on results of the three clocks will be reported in this order, see also Table 2).

**Figure 2:**
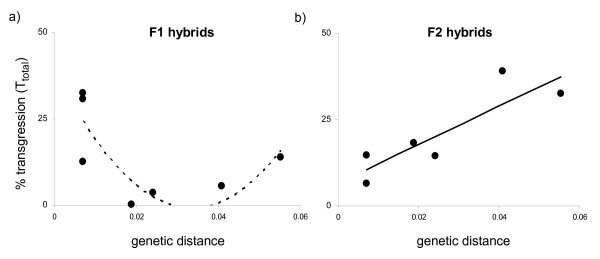
**Total amount of transgression (*T*_*total*_) observed in interspecific a) F1 hybrids and b) F2 hybrids as a function of genetic distance (uncorrected p-distance) between the parental species**. Regression lines are from quadratic (a) and linear (b) model fitting. The solid line indicates a (significant) linear relationship in F2 hybrids, the dotted line indicates a (nonsignificant) quadratic relationship in F1 hybrids.

In F1 hybrids, testing genetic distance as a predictor for the total amount of transgression (*T*_*total*_) resulted in a u-shaped relationship although this was not significant (quadratic regression: R^2 ^(adjusted) = 0.52, F_2,6 _= 4.21, p = 0.104; Figure 2a). Large amounts of transgression were observed in hybrids between both closely and distantly related crosses (13-33% in closely related crosses, 14% in distant crosses) with a near absence of transgression (0.1-6%) in crosses of intermediate genetic distance.

In F2 hybrids, transgression significantly increased with genetic distance (linear regression: R^2 ^= 0.78, F_1,5 _= 12.29, p = 0.025; Figure 2b) with a minimum of 6% transgression in closely related crosses and a maximum of 39% transgression in distant crosses.

### Transgressive segregation as a function of phenotypic differentiation

**Figure 3:**
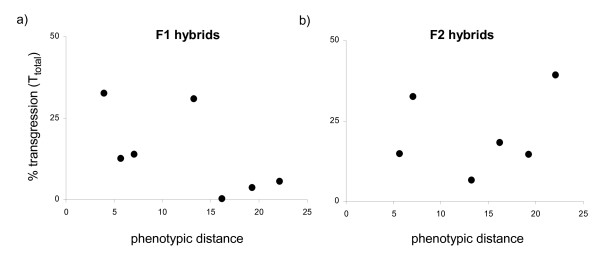
**Total amount of transgression (*T*_*total*_) observed in interspecific a) F1 hybrids and b) F2 hybrids as a function of the phenotypic distance (Mahalanobis distance calculated from 15 geometric morphometric landmarks) between the parental species**. No significant relationships were found.

According to our prediction, transgression should decrease as a function of phenotypic dissimilarity between the parental species. Testing phenotypic distance (calculated as Mahalanobis distance) as a predictor for the amount of transgression (*T*_*total*_) did not result in a significant relationship in F1 hybrids (linear regression: R^2 ^= 0.38, F_1,6 _= 3.06, p = 0.140; Figure 3a) nor in F2 hybrids (linear regression: R^2 ^= 0.05, F_1,5 _= 0.21, p = 0.674; Figure 3b).

We further tested if phenotypic and genetic divergence between the parental species were correlated. Although we found a positive trend, the relationship was not significant (logarithmic regression: R^2 ^= 0.22, F_1,6 _= 1.45; p = 0.28) due to one outlying data point (*P. nyererei × A. calliptera*, cross 7; the exclusion of this outlier resulted in a strong positive correlation: R^2 ^= 0.81, F_1,5 _= 17.32; p = 0.014).

## Discussion and Conclusion

Studies on interspecific animal and plant hybrids have demonstrated that hybridization frequently gives rise to phenotypic novelty. One source of such novelty that may facilitate adaptive evolution is transgressive segregation which refers to the occurrence of hybrid phenotypes that express trait values exceeding the phenotypic range of both parental species combined [25,26]. Evidence supporting the notion that transgressive ecomorphological and ecophysiological trait values can generate functional novelty that permits colonizing previously underutilized peaks on a fitness landscape comes from detailed work on *Helianthus *sunflowers. This work conclusively demonstrated how transgression in key ecological traits can allow hybrids to invade an ecologically and spatially distinct niche and in turn escape the homogenizing effects of gene flow from parental species [10,13,44,45].

Here, we used African haplochromine cichlid fish from two large adaptive radiations (Lake Victoria, Lake Malawi) and two riverine species that are related to the radiations (*A. calliptera*, *A. burtoni*) to test if the amount of transgression in interspecific hybrids increased as a function of genetic distance between species. We made seven different cross types from different species combinations representing five different genetic distances, covering absolute divergence times of between a few thousand years to 2.7/3.8/7.4 million years depending on the molecular clock used (see Table 2). One of these crosses (*A. calliptera *× *A. burtoni*) was between two riverine species of the genus that was ancestral to the two large African radiations. Using geometric morphometrics on the multi-trait phenotypes we quantified and compared shape variation in F1 and F2 generation hybrids and in the two corresponding homospecific control crosses.

We predicted to observe an increase of transgression with increasing genetic crossing distance in both F1 and F2 hybrids. In F1 hybrids, the increase may be predicted to be less steep than in F2 hybrids because (besides epistatic interactions) only dominant genetic effects can contribute to complementary gene action, while in F2 hybrids, additive genetic effects also contribute. We found that transgressive segregation was frequent and that extreme trait values were produced in each F1 and F2 cross type. The amount of transgression observed in F2 hybrids increased linearly with time since speciation (Figure 2b) confirming our prediction. However, in the F1 hybrids, large amounts of transgression were expressed in hybrids between both closely and distantly related species but transgression was nearly absent in hybrids of parents with intermediate genetic distances, resulting in a u-shaped relationship between transgression and divergence time (Figure 2a).

While the increase of transgression in F1 hybrids of distant crosses can be explained by a higher frequency of epistatic interactions and dominant genetic effects, the large amount of transgression observed in F1 hybrids of closely related species was unexpected. Models of complementary gene action in transgressive segregation usually assume that parental species are fixed for QTL alleles. It is possible that the closely related species in our experiment produced transgressive F1 progeny because the parents were heterozygous at some QTL. This is possible because all our closely related species had sympatric distribution ranges within Lake Victoria where interspecific hybridization may occasionally occur [46]. Alternatively, overall increased hybrid vigour, accompanying increased average heterozygosity in the F1 hybrid generation, may have led to larger and hence transgressive trait values in more vigorous individuals of crosses between closely related species. Generally, the relationship between offspring vigour and the genetic distance between their parents is predicted to be dome-shaped (with a left-shifted mode) confirmed by both experimental [47-52] and theoretical work [53,54]. This is thought to be due to the effects of inbreeding depression at small distances and the effects of genetic incompatibilities, the break-up of co-adapted gene complexes, epistatic interactions and underdominance (heterozygote disadvantage) at larger distances. Hence, the fitness peak will typically reside in the region representing intraspecific between-population matings. If, however, speciation was recent - as in the case of rapidly radiating species flocks - the intrinsically determined fitness peak (disregarding extrinsic, ecologically-determined fitness) may well be shifted to overlap with the interspecific region. On this note, it would be useful to determine the genetic distance where the increasing effects of genetic incompatibilities and the decreasing heterosis effects typically cancel out, to assess if this may have caused the depression in the amount of transgression at intermediate genetic distances in F1 hybrids observed in our experiment.

We further found that the degree of phenotypic differentiation of the parental species in our experiment was not predicted by genetic distance. This is in agreement with a recent comparative genomic analysis of Lake Malawi cichlids showing that cichlid species can be phenotypically and behaviorally diverse while showing levels of genome-wide differentiation not larger than typically observed between subdivided populations of the same species [55]. Because phenotypic distance, in contrast to genetic distance, is predicted to have a negative effect on the occurrence of transgression, the effects of both variables can theoretically cancel out. We thus tested if transgression was also a function of the increasing phenotypic dissimilarity between species. Contrary to our prediction, the amount of transgression in both F1 and F2 hybrids was independent of phenotypic differentiation (Figure 3). It is hence unlikely that the counteracting effects of phenotypic divergence in our experiment compromised the effect of genetic distance.

Except for the three species crosses representing the lowest end of the genetic distance gradient in our experiment, most of the species we used are allopatric in the wild (crosses 4-7, Table 1; note that even though *A. calliptera *occurs in the same lake with *P. taeniolatus *and *M. estherae *it has little habitat overlap with either). They presumably acquired divergent phenotypes as a result different selection regimes in different environments, with perhaps contributions of drift, rather than due to consistent and strong disruptive selection on the same traits, which would have purged many of the antagonistic allelic effects within QTLs. It is hence likely that alleles of opposing signs were preserved during the divergence of even the phenotypically most divergent species in our experiment, resulting in frequent opportunity for complementary gene action in their hybrids. The latter may explain why the amount of transgression is not a function of phenotypic divergence in our data set. Our experimental design is not suitable to test the effect of a gradually increasing disruptive selection coefficient on the amount of transgression but this relationship is certainly worthwhile to be investigated in future experiments.

All factors considered it seems plausible that the observed increase in transgression with genetic distance in F2 hybrids is mainly the result of an increasing opportunity for complementary gene action and epistasis in hybrids between genetically more distant lineages. This is probably due to an increasing number of QTLs for which the diverging species fix alleles with opposite signs, providing more frequent opportunity for transgression in interspecific hybrids (note that we do not refer here to consistent directional selection which would fix positive signs across all QTLs in one species and all negative signs in the other species).

Implications of the observed positive relationship between genetic distance and transgression are particularly interesting where hybridization between distantly related lineages has taken place at the onset of rapid adaptive radiations. Traces of ancient hybridization in phylogenetic reconstructions of several plants and animal radiations suggest that genetic exchange between at least two distantly related lineages occurred at the onset of radiations, and may have acted as a catalyst for the rapid phenotypic diversification of these groups [15,17,19,56,57]. The largest genetic distance between species in our experiment represents similar divergence times (2.7/3.8/7.4 my) to those estimated for the hypothesized, anciently hybridized ancestors of two major cichlid radiations (Lake paleo-Makgadikgadi [15], Lake Victoria [16]). In fact, the *Astatotilapia calliptera *× *A. burtoni *cross could be considered a simulation of what effect hybridization between the ancestors of these radiations would have had on phenotypic variation. These two species are phenotypically and ecologically very similar to the putative ancestors of the Lake Victoria region adaptive radiation [16].

It is worth mentioning that many of the hybrids we obtained phenotypically resemble other species known from the cichlid radiations, an observation made before on other cichlid hybrid phenotypes generated in the laboratory [27,58]. For example, hybrids between *P. chilotes *and *P. nyererei *resembled the Lake Victoria species *Haplochromis *sp. "thickskin" in overall body and head morphology, whereas hybrids between *A. calliptera *and *P. nyererei *resembled another Lake Victoria species (*Pundamlia *sp. "yellow azurea") in coloration and body shape. These observations make it indeed plausible that hybridization between divergent genomes has contributed to the unusually rapid rates of phenotypic evolution in haplochromine cichlids. Transgressive segregation potentially increases the working surface for selection well beyond that provided by standing genetic variation within just two generations. It can thus provide rapid momentum to the adaptive diversification of a group under multifarious selection by cutting the waiting time to new mutations. Some hybrid species have indeed been shown to establish in new ecological niches in very few generations [28,59]. If transgressive segregation was an important contributor to the volume and extent of phenotypic diversification during adaptive radiations [19,27,60], variation in the genetic architecture between lineages (which can be either conducive or obstructive to complementary gene action) might cause variation in the rates of adaptive radiation observed between lineages. This hypothesis is speculative at this moment and awaits rigorous testing.

## Methods

### Producing hybrids

Crosses used nine species of haplochromine cichlids from Lake Victoria, Lake Malawi and East African rivers (Table 1), representing different, ecologically specialized groups. Among them were a rock-dwelling planktivore (*Pundamilia nyererei*), an insect larvae picker (*Paralabidochromis chilotes*), two trophic generalists (*Pundamilia pundamilia, Paralabidochromis rockkribensis*), rock-dwelling algae scrapers (*Neochromis omnicaeruleus*, *Metriaclima estherae*), algae suckers (*Protomelas taeniolatus*), and two habitat generalists (*Astatotilapia calliptera*, *Astatotilapia burtoni*) [61,62]. All species are female mouthbrooders and inhabit shallow waters (1-10 m in depth).

All parental individuals used for making hybrid crosses were derived from laboratory populations bred from fish collected in Lake Victoria and Lake Malawi and maintained in the large fish breeding facility at EAWAG, Switzerland.

Seven different F1 hybrid cross types were obtained by populating aquaria (100 × 40 × 40 cm) with five to twenty females of one species and one heterospecific male. Subsequently, F2 hybrids were bred from different males and females of six different F1 hybrid cross types (one F1 cross type, *P*.*rockkribensis *× *P*.*pundamilia*, could not be bred further due to space constraints. However, the genetic distance of this pair is represented by two other cross types in the experiment; Table 2). No fish was used to produce more than one hybrid family.

Experimental tanks were part of a large water recirculation system, light regime was 12L:12D and water temperature was kept constant at 24 - 26°C. All animals were fed the same food (dry food every day, and a blend of shrimps, peas and *Spirulina *powder two times a week) allocated in equal amounts every day, and were raised to 180 days in age. At 180 days almost all individuals had reached sexual maturity. Further information regarding breeding and maintenance is given elsewhere [63].

### Measuring transgressive shape segregation using geometric morphometrics

**Figure 4:**
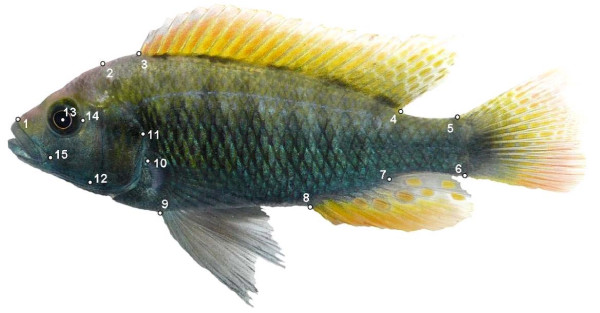
**F1 hybrid individual between the two African haplochromine cichlid species *Pundamilia nyererei *and *Astatotilapia calliptera***. Numbers label the 15 landmarks used for geometric morphometric analysis of body shape variation in interspecific hybrids. 1) Anterior tip of maxilla, 2) junction of head and dorsal scales, 3) anterior insertion of dorsal fin, 4) posterior insertion of dorsal fin, 5) dorsal junction of caudal fin and caudal peduncle, 6) ventral junction of caudal fin and caudal peduncle, 7) posterior insertion of anal fin, 8) anterior insertion of anal fin, 9) anterior insertion of pelvic fin, 10) dorsal insertion of pectoral fin, 11) posterior reach of operculum, 12) lower margin of preopercule, 13) centre of the eye, 14) anterior insertion of the preopercule, and 15) anterior reach of the premaxillary groove.

All hybrids and the corresponding homospecific individuals were photographed at the age of 180 (± 1) days. Pictures were taken of the left side of the live fish in a transparent photo cuvette with a scale for size calibration. Geometric morphometric analysis was performed on the x-y coordinates of 15 landmarks placed on the photographs (Figure 4) using tpsDig version 2.10, [64]. To reduce noise introduced through variation in position, orientation and size, this non-shape variation was mathematically removed using generalized procrustes analysis (GPA) [65,66]. GPA superimposes landmark configurations in that it minimizes the sum of squared distances between corresponding landmarks by scaling, translating and rotating specimens onto a mean consensus configuration calculated from all specimens. Thin-plate spline (TPS) procedure was then applied to obtain partial warps using tpsRelw version 1.45 [67]. Partial warps estimate the minimum bending energy needed to deform an infinitely thin metal plate (i.e. the landmark configuration of an individual fish) to adopt the shape of another landmark configuration (i.e. the consensus configuration among all the fish) while being constrained at particular points (i.e the landmarks). The total deformation of the spline can be broken down into geometrically orthogonal components in a Cartesian coordinate system (i.e. the partial warps) to describe the amount of stretching, bending and twisting necessary to superimpose the coordinates of all specimens onto the consensus shape. Each individual then has a weight for the x- and y- components of each partial warp.

All subsequent analyses were performed in JMP 7.0 [68]. Partial warp weights were regressed against size and residuals of these were used for all further analysis to remove potential allometric size effects. Residuals were entered into principal component analysis (PCA) to identify the major axes of shape variation, which is also referred to as relative warp analysis [69]. We extracted all principal components that explained more than 5% of the variance in the data set (between 4 and 6 components, the number of PCs used per cross type is shown in Additional file 3).

**Figure 5:**
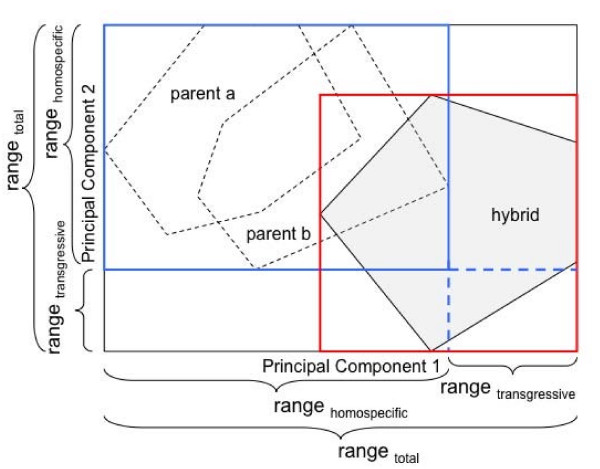
**Schematic drawing of the variables used in equation 1 (see Methods) to quantify the amount of transgression found in hybrid crosses**. Note that this was done for all relevant PCs, not only for PC1 and PC2.

The amount of transgression (*T*_*PCi*_) occurring along a principal component axis (PC_*i*_) was calculated as

where *range*_*total *_is the total phenotypic range between the largest and smallest observation of all hybrid and homospecific individuals of a particular cross type, and *range*_*homospecific *_represents the phenotypic range including only homospecific individuals of that cross type. The numerator hence stands for the transgressive portion of the hybrid range (*range*_*trans*_; for a schematic drawing of the variables see Figure 5). We then calculated the sum of transgression found along all PCs to obtain the total amount of transgression (*T*_*total *_which can be larger than 100%). This was done in a weighted averaging procedure, where *T*_*PCi *_was multiplied with the percentage variance explained by that PC.

To test the effect of increasing genetic distance on the amount of transgression (*T*_*total*_) we regressed *T*_*total *_against genetic distance using linear regression models. Normal distribution of variables was confirmed with Shapiro-Wilk tests.

To test whether families within cross types differed in their phenotype distribution, we used MANOVA with family as factor and all relevant PCs as response variables. This analysis was performed on both hybrids and homospecific crosses.

### Measuring genetic distance and divergence time

Genetic distances were estimated for every species pair used for making hybrid crosses by calculating uncorrected p-distances from D-loop sequences downloaded from NCBI GenBank (http://ncbi.nlm.nih.gov/Genbank/; accession numbers of all sequences can be found in Additional file 4). All available sequences of every species were included for calculating genetic distances. For six species no sequences were available (*P. pundamilia, P. nyererei, N. omnicaeruleus, P. rockkribensis, P. chilotes, M. estherae*). In these cases we used sequences from a very closely related species. This was in all cases justified because both species (experimental and substitute) belonged to the same clade within which mitochondrial DNA haplotype sorting is highly incomplete (i.e. the radiation of Lake Victoria and a clade of the Lake Malawi Mbuna). Sequences were aligned in ClustalW [70] using the pairwise alignment algorithm and alignments were manually controlled and improved locally. Genetic distances were calculated in MEGA 4 [71]. Where multiple sequences were available, we took the average of all possible pairwise interspecific p-distances (e.g. [72,73]. To correct comparisons between species for the variation occurring within species, mean intraspecific genetic distances (the mean of the two species means) were subtracted from mean interspecific distances [73,74].

Genetic distances were converted into absolute times of divergence using two different non-linear relaxed molecular clocks (one calibrated using the cichlid fossil record and recent geological events and the other using the fragmentation of Gondwanaland and recent geological events [75]). In addition, we used an internally calibrated linear clock that has been widely used in cichlid phylogeography [76]. We note that there is increasingly wide support for the Gondwana fragmentation clock [77].

### Measuring phenotypic shape divergence

Phenotypic shape divergence was quantified by measuring the mean of all Mahalanobis distances between individuals of any two species. As variables we used all principal components (from a PCA including both parental species) that explained more than 5% of the variance. Distances were then averaged to obtain a measure of the overall phenotypic dissimilarity of any two parental species. To correct comparisons between species for the variation occurring within species, mean intraspecific phenotypic distances (the mean of the two species means) were subtracted from mean interspecific distances.

To test the effect of increasing phenotypic distance on the amount of transgression, *T*_*total *_was regressed against phenotypic distance using linear regression models. Normality of distribution was confirmed with Shapiro-Wilk tests.

## Authors' contributions

O. Seehausen conceived of the study. R. B. Stelkens and O. Seehausen designed the study. R. B. Stelkens bred and processed the F1 generation hybrids. R. B. Stelkens and C. Schmid bred the F2 generation hybrids. R. B. Stelkens, C. Schmid and O. Selz processed the F2 generation hybrids. R. B. Stelkens carried out sequence alignment. R. B. Stelkens and C. Schmid performed the statistical analysis. R. B. Stelkens and O. Seehausen wrote the manuscript. All authors read and approved the final manuscript.

## Supplementary Material

Additional file 1 **Shape analysis of interspecific F1 hybrids using principal components**. Figure showing results of principal component analyses using geometric morphometrics data to quantify the amount of transgression in shape of interspecific F1 hybrids of haplochromine cichlids. Click here for file

Additional file 2 **Transgression and shape variance explained per PC axis**. Table showing the amount of transgression and the shape variance explained per PC axis for both F1 and F2 hybrid crosses. Click here for file

Additional file 3 **Family effects on shape variance**. Table showing test results from MANOVA with family as factor and PC scores as response variables. Click here for file

Additional file 4 **Genbank accession numbers**. Table showing NCBI Genbank accession numbers of D-loop sequences used for calculations of genetic distances. Asterisks indicate cases where no or insufficient sequences were available for the species used in the experiments. Click here for file
