# Supplementary material for: Phenotypic novelty in experimental hybrids is predicted by the genetic distance between species of cichlid fish
Source: BMC Evol Biol. 2009 Dec 4;9:283. doi: 10.1186/1471-2148-9-283 (PMC2796671; doi:10.1186/1471-2148-9-283)
Supplement: Additional file 1 — Shape analysis of interspecific F1 hybrids using principal components. Figure showing results of principal component analyses using geometric morphometrics data to quantify the amount of transgression in shape of interspecific F1 hybrids of haplochromine cichlids. [file 1471-2148-9-283-S1.PDF]

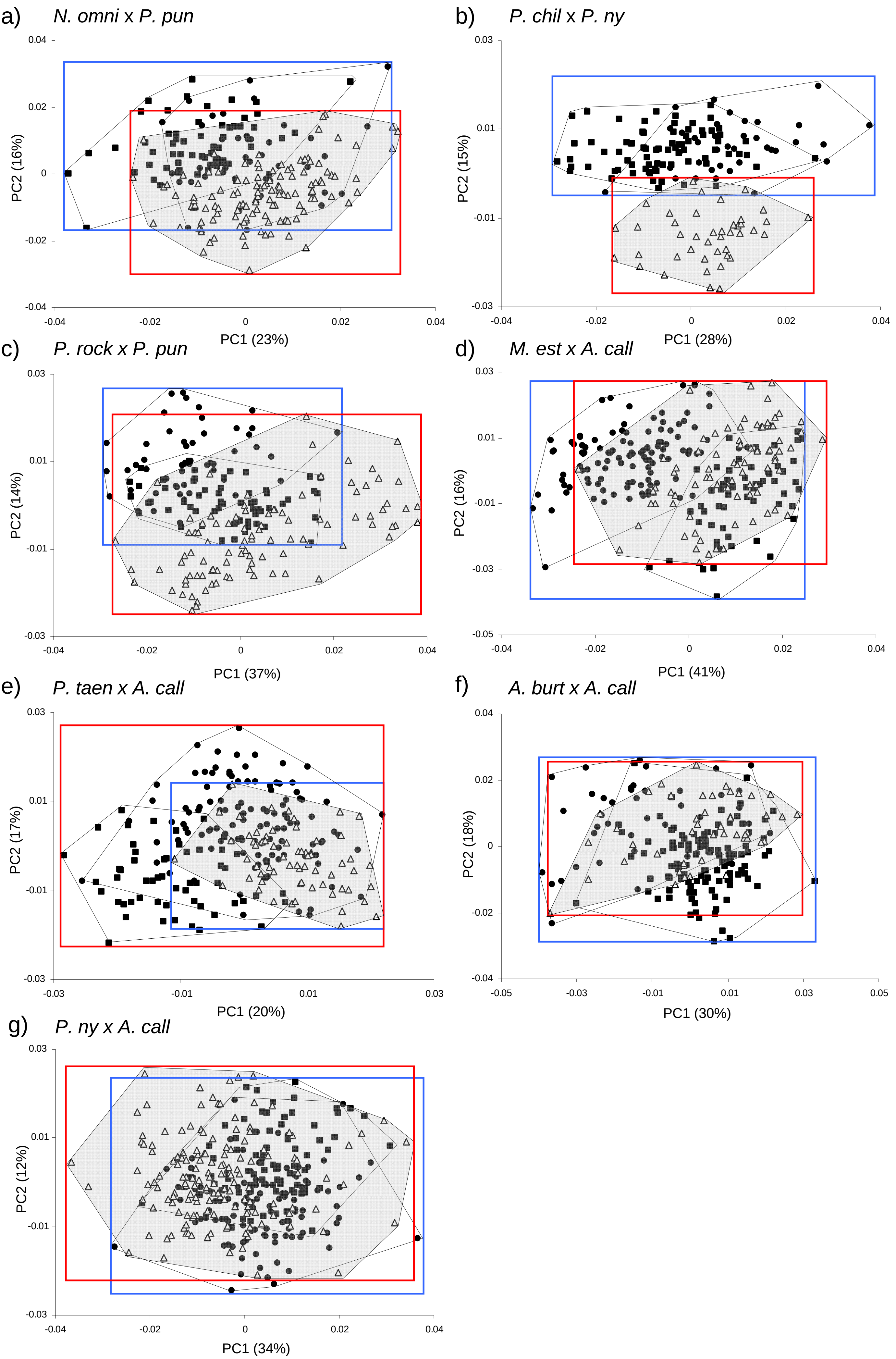
a) N. omni x P. pun
0.04
0.02
0
-0.02
-0.04
PC2 (16%)
-0.04
-0.02
0
0.02
0.04
PC1 (23%)
b) P. chil x P. ny
0.03
0.01
-0.01
-0.03
PC2 (15%)
-0.04
-0.02
0
0.02
0.04
PC1 (28%)
c) P. rock x P. pun
0.03
0.01
-0.01
-0.03
PC2 (14%)
-0.04
-0.02
0
0.02
0.04
PC1 (37%)
d) M. est x A. call
0.03
0.01
-0.01
-0.03
-0.05
PC2 (16%)
-0.04
-0.02
0
0.02
0.04
PC1 (41%)
e) P. taen x A. call
0.03
0.01
-0.01
-0.03
PC2 (17%)
-0.03
-0.01
0.01
0.03
PC1 (20%)
f) A. burt x A. call
0.04
0.02
0
-0.02
-0.04
PC2 (18%)
-0.05
-0.03
-0.01
0.01
0.03
0.05
PC1 (30%)
g) P. ny x A. call
0.03
0.01
-0.01
-0.03
PC2 (12%)
-0.04
-0.02
0
0.02
0.04
PC1 (34%)

Additional file 1: Results of principal component analyses using geometric morphometrics data to quantify the amount of transgression in shape of interspecific hybrids of haplochromine cichlids. Graphs show the distribution in morphospace of seven different F1 hybrid crosses and the corresponding homospecific crosses of species pairs with increasing genetic distance from smallest (a, b, c) to largest distance (g). Abbreviations of species names correspond to Table 2. Every data point represents one individual. Filled symbols indicate parental species, triangles indicate F1 hybrids. Blue squares encompass the phenotype range of the combined parental species; red squares represent the phenotype range of F1 hybrids. The percentage of variance explained by principal component 1 and 2 are shown in brackets. Note that the visualization of transgression is restricted to the first two axes of shape variation here, which is not (or not entirely) representative of the total amount of transgression found per cross type.
