# Supplementary material for: Phenotypic novelty in experimental hybrids is predicted by the genetic distance between species of cichlid fish
Source: BMC Evol Biol. 2009 Dec 4;9:283. doi: 10.1186/1471-2148-9-283 (PMC2796671; doi:10.1186/1471-2148-9-283)
Supplement: Additional file 2 — Transgression and shape variance explained per PC axis. Table showing the amount of transgression and the shape variance explained per PC axis for both F1 and F2 hybrid crosses. [file 1471-2148-9-283-S2.DOC]

Additional file 2: The amount of transgression and the shape variance explained per PC axis in both F1 and F2 hybrid crosses.

| **cross type** | **Species crossed** | **TS on PC1 (%)** | **variance**  **(%)** | **TS on PC2 (%)** | **variance**  **(%)** | **TS on PC3 (%)** | **variance**  **(%)** | **TS on PC4 (%)** | **variance**  **(%)** | **TS on PC5 (%)** | **variance**  **(%)** | **TS on PC6 (%)** | **variance**  **(%)** |
| --- | --- | --- | --- | --- | --- | --- | --- | --- | --- | --- | --- | --- | --- |
|  | **F1 hybrids** |  |  |  |  |  |  |  |  |  |  |  |  |
| 1 | *N. omni x P. pun* | 20.62 | 31.95 | 24.20 | 24.65 | 0.00 | 15.71 | 0.00 | 11.22 | 0.00 | 9.12 | 0.00 | 7.35 |
| 2 | *P. chil x P. ny* | 0.00 | 30.55 | 134.46 | 22.78 | 0.00 | 17.84 | 0.00 | 12.01 | 0.00 | 9.35 | 1.80 | 7.47 |
| 3 | *P. rock x P. pun* | 55.79 | 36.40 | 24.49 | 19.51 | 18.36 | 12.95 | 43.89 | 11.44 | 0.00 | 10.31 | 0.00 | 9.38 |
| 4 | *M. est x A. call* | 0.00 | 45.32 | 0.00 | 31.31 | 0.98 | 13.86 | 0.00 | 9.51 | - | - | - | - |
| 5 | *P. taen x A. call* | 0.00 | 27.60 | 0.00 | 23.83 | 3.12 | 16.01 | 0.00 | 13.22 | 31.23 | 10.30 | 0.00 | 9.04 |
| 6 | *A. burt x A. call* | 0.00 | 42.19 | 0.00 | 25.85 | 38.60 | 12.89 | 6.03 | 10.03 | 0.00 | 9.04 | - | - |
| 7 | *P. ny x A. call* | 14.19 | 32.82 | 27.26 | 24.15 | 16.69 | 14.22 | 0.00 | 11.31 | 2.79 | 10.17 | 0.00 | 7.33 |
|  | **F2 hybrids** |  |  |  |  |  |  |  |  |  |  |  |  |
| 1 | *N. omni x P. pun* | 27.44 | 34.04 | 22.42 | 24.04 | 0.00 | 18.72 | 0.00 | 12.56 | 0.00 | 10.64 |  |  |
| 2 | *P. chil x P. ny* | 8.46 | 38.03 | 0.00 | 20.65 | 13.85 | 13.85 | 8.01 | 11.63 | 4.33 | 8.17 | 0.00 | 7.67 |
| 3 | *M. est x A. call* | 28.36 | 53.37 | 0.00 | 20.72 | 22.66 | 10.50 | 0.55 | 8.78 | 9.56 | 6.64 |  |  |
| 4 | *P. taen x A. call* | 26.28 | 50.95 | 0.00 | 18.08 | 8.05 | 12.59 | 0.00 | 10.29 | 0.00 | 8.10 |  |  |
| 5 | *A. burt x A. call* | 54.37 | 54.73 | 14.91 | 19.80 | 37.97 | 15.88 | 3.50 | 9.59 |  |  |  |  |
| 6 | *P. ny x A. call* | 60.93 | 0.61 | 4.43 | 0.04 | 7.56 | 0.08 | 5.20 | 0.05 | 1.85 | 0.02 |  |  |
