# Supplementary material for: Phenotypic novelty in experimental hybrids is predicted by the genetic distance between species of cichlid fish
Source: BMC Evol Biol. 2009 Dec 4;9:283. doi: 10.1186/1471-2148-9-283 (PMC2796671; doi:10.1186/1471-2148-9-283)
Supplement: Additional file 3 — Family effects on shape variance. Table showing test results from MANOVA with family as factor and PC scores as response variables. [file 1471-2148-9-283-S3.DOC]

Additional file 3: Test results from MANOVA with family as factor and PC scores as response variables. For the number of families per cross type see Table 2.

| **cross type** | **Species crossed** | **PC1** | | **PC2** | | **PC3** | | **PC4** | | **PC5** | | **PC6** | |
| --- | --- | --- | --- | --- | --- | --- | --- | --- | --- | --- | --- | --- | --- |
|  | **F1 hybrids** | ***F*** | ***p*** | ***F*** | ***p*** | ***F*** | ***p*** | ***F*** | ***p*** | ***F*** | ***p*** | ***F*** | ***p*** |
| 1 | *N. omni x P. pun* | 20.30 | < 0.001 | 9.15 | < 0.001 | 6.91 | < 0.001 | 7.15 | < 0.001 | 2.52 | 0.061 | 0.75 | 0.525 |
| 2 | *P. chil x P. ny* | 0.02 | 0.892 | 6.07 | 0.018 | 2.43 | 0.127 | 0.14 | 0.713 | 1.53 | 0.223 | 3.91 | 0.055 |
| 3 | *P. rock x P. pun* | 46.50 | < 0.001 | 98.32 | < 0.001 | 0.68 | 0.507 | 4.68 | 0.011 | 0.55 | 0.578 | 0.63 | 0.532 |
| 4 | *M. est x A. call* | 7.35 | < 0.001 | 9.70 | < 0.001 | 1.91 | 0.080 | 1.26 | 0.280 | - | - | - | - |
| 5 | *P. taen x A. call* | 0.36 | 0.549 | 2.60 | 0.112 | 0.40 | 0.528 | 4.24 | 0.044 | 3.58 | 0.063 | 0.10 | 0.759 |
| 6 | *A. burt x A. call* | 10.09 | < 0.001 | 1.13 | 0.348 | 0.30 | 0.828 | 23.24 | <.0001 | 0.21 | 0.890 | - | - |
| 7 | *P. ny x A. call* | 3.30 | 0.003 | 7.09 | < 0.001 | 8.48 | < 0.001 | 4.07 | < 0.001 | 2.24 | 0.034 | 2.44 | 0.022 |
|  | **F2 hybrids** | ***F*** | ***p*** | ***F*** | ***p*** | ***F*** | ***p*** | ***F*** | ***p*** | ***F*** | ***p*** | ***F*** | ***p*** |
| 1 | *N. omni x P. pun* | 9.77 | < 0.001 | 12.50 | < 0.001 | 1.29 | 0.295 | 5.62 | 0.003 | 0.80 | 0.502 | - | - |
| 2 | *P. chil x P. ny* | 3.17 | 0.022 | 1.04 | 0.398 | 1.29 | 0.286 | 7.41 | <.0001 | 11.24 | <.0001 | 2.80 | 0.036 |
| 3 | *M. est x A. call* | 2.36 | 0.013 | 5.99 | < 0.001 | 1.75 | 0.076 | 3.00 | 0.002 | 3.20 | 0.001 | - | - |
| 4 | *P. taen x A. call* | 1.76 | 0.122 | 1.21 | 0.315 | 4.10 | 0.002 | 1.21 | 0.314 | 2.23 | 0.052 | - | - |
| 5 | *A. burt x A. call* | 2.88 | 0.030 | 6.05 | < 0.001 | 10.24 | < 0.001 | 10.96 | < 0.001 | - | - | - | - |
| 6 | *P. ny x A. call* | 2.87 | 0.013 | 4.50 | 0.001 | 0.21 | 0.981 | 4.05 | 0.001 | 2.78 | 0.015 | - | - |
