# Supplementary material for: Phenotypic novelty in experimental hybrids is predicted by the genetic distance between species of cichlid fish
Source: BMC Evol Biol. 2009 Dec 4;9:283. doi: 10.1186/1471-2148-9-283 (PMC2796671; doi:10.1186/1471-2148-9-283)
Supplement: Additional file 4 — Genbank accession numbers. Table showing NCBI Genbank accession numbers of D-loop sequences used for calculations of genetic distances. Asterisks indicate cases where no or insufficient sequences were available for the species used in the experiments. [file 1471-2148-9-283-S4.DOC]

Additional file 4: NCBI Genbank accession numbers of D-loop sequences used for calculations of genetic distances. Asterisks indicate cases where no or insufficient sequences were available for the species used in the experiments.

| **species** | **Genbank accession numbers** |
| --- | --- |
| ** Pundamilia pundamilia/nyererei*  ** Neochromis omnicaeruleus*  ** Paralabidochromis rockkribensis/chilotes* | AF213528, AF213544, AF213545, AY930005, AY930006 *(Neochromis nigricans)*  AF213540, AF213539, AF213525 *(Paralabidochromis chilotes)*  AF213546, AF213548, AF213547, AF213529 *(Paralabidochromis plagiodon )* |
| ** Metriclima estherae* | AY930025 (*Metriaclima. zebra*),  AF213620, AY911810, AY911811, AY911812 (*Metriaclima callanois*) |
| *Astatotilapia calliptera* | AF298938, AY911722, AY929977, AF298940, AF298939, AF298941, AY911723 |
| *Astatotilapia burtoni* | AY929999, AF298905, AY929955, AF298906, AY930000, AY930001, AF298904 |
| *Protomelas taeniolatus* | AF298963, AY913942, EF6475464 |
